# Comparative analysis of glycated haemoglobin, fasting blood glucose and haematological parameters in Type-2 diabetes patients

**DOI:** 10.1186/s13104-023-06520-x

**Published:** 2023-10-05

**Authors:** Samuel Antwi-Baffour, Benjamin Tetteh Mensah, Dorinda Naa Okailey Armah, Samira Ali-Mustapha, Lawrence Annison

**Affiliations:** 1 Department of Medical Laboratory Sciences, School of Biomedical and Allied Health Sciences, College of Health Sciences, University of Ghana, Korle-Bu, P. O. Box KB 143, Accra, Ghana; 2 Department of Medical Laboratory Technology, School of Medical Sciences, Accra Technical University, Accra, Ghana; 3 Department of Maternal and Child Health, School of Nursing, University of Ghana, Legon, Ghana

**Keywords:** Diabetes mellitus, Glycated haemoglobin, Haematological, Glucose

## Abstract

**Objective:**

Diabetes remains a major health problem, and Glycated hemoglobin (HBA1c) and fasting blood glucose (FBG) levels play important roles in its management. Also, chronic hyperglycemia coupled with high HBA1c levels impact inflammation and may alter haematological parameters in diabetes. Hence, the need to assess and correlate HBA1c and FBG levels with selected haematological parameters in patients with type-2 diabetes mellitus as the main objective of this study. The study was cross-sectional involving 384 participants. Five milliliters of blood was collected from each participant and analyzed for HBA1c, FBG levels and full blood count which were correlated statistically.

**Results:**

From the data obtained and analyzed, there were statistically significant correlations between HBA1c and neutrophil count (p < 0.013), plateletcrit (p < 0.036), mean platelet volume (p < 0.019) and platelet distribution width (p < 0.002). There were also significant differences in FBG (p < 0.014), neutrophil count (p < 0.029), red cell distribution width (p < 0.046), mean platelet volume (p < 0.032) and platelet distribution width (p < 0.013) between diabetes patients with HBA1c less than 7.0% and HBA1c more than or equal to 7.0%. The outcome of the study indicates significant correlation of HBA1c with selected haematological parameters. This could make routine haematological parameters a cost-effective means of predicting poor glucose control in diabetes mellitus patients.

**Supplementary Information:**

The online version contains supplementary material available at 10.1186/s13104-023-06520-x.

## Introduction

Diabetes is a metabolic disorder of carbohydrate metabolism which result in persistent hyperglycemia due to either insulin deficiency, insulin resistance or both [1]. According to the Diabetes Atlas (2017), an estimated 425 million people were living with diabetes in 2017 worldwide with the global diabetes prevalence standing at 8.8% [2]. Hyperglycemia in diabetes, especially uncontrolled diabetes, over time, results in serious damage to multiple organs such as kidneys, eyes, nerves, blood vessels and the heart, collectively referred to as microvascular and macrovascular complications of diabetes in the body [3, 4]. Thus, blood glucose monitoring and regulation is very crucial in improving prognosis in diabetes [4].

Diabetes mellitus can be diagnosed by measuring fasting blood glucose (FBG), random blood glucose, Glycated haemoglobin (HBA1c) levels or by performing an oral glucose tolerance test (OGTT) according to the WHO [5]. However, HBA1c and FBG are key markers in the management of patients with diabetes. This is because HBA1c levels represent the integrated blood glucose concentration over the preceding 8 to 12 weeks [6].

Again, inflammation and altered platelet functions have been found to be evident in diabetes, thus some researchers have proposed particular haematological indices to be independent predictors of diabetes [7–9]. Some researchers have also found haematological indices to be useful indicators of vascular complications and glucose control in diabetes patients [10]. In the Ghanaian setting, data on haematological parameters and various facets of diabetes mellitus remain scanty. Only limited amount of studies have reported on Glycated haemoglobin, fasting blood glucose and haematological parameters in patients with diabetes in Ghana. But a correlation between the three parameters has not been studied extensively, hence the need for this study.

## Methods

### Aim, design and setting of the study

The aim of this study was to correlate Glycated haemoglobin levels and fasting blood glucose with selected haematological parameters in patients with diabetes mellitus. A cross-sectional study design was employed for this study at the Korle-Bu teaching hospital (KBTH).

### Inclusion criteria

The study targeted patients of all age groups diagnosed with type-2 diabetes mellitus and receiving treatment at the diabetes clinic of the KBTH.

### Exclusion criteria

The study excluded patients who did not have type-2 diabetes and those with diabetes who were pregnant or had other conditions such as cancer, sickle cell disease or bone marrow disorders.

### Data collection procedure

#### Sample collection

Five milliliters of venous blood were collected from the antecubital fossa using standard phlebotomy procedures. Three milliliters of the blood were transferred into an EDTA anticoagulant tube and two milliliters into fluoride oxalate anticoagulant tube for analysis.

#### Sample analysis

##### *HbA1c determination*

The levels of HBA1c were determined on an automated Vitros chemistry analyzer (Ortho Clinical Diagnostics, U.S). For each sample, patient information was input into the analyzer and blood samples in EDTA were placed accordingly into small sample cups. The sample cups were arranged in the same order on labelled sample trays according to the program of the analyzer. They were then loaded onto the analyzer to run.

##### *FBG determination*

Fasting blood glucose concentrations were also measured on an automated Vitros chemistry analyzer. Samples in fluoride oxalate were centrifuged at 3000 rpm for 2 min to separate the plasma from the blood cells. Each patient plasma was poured into small sample cups and tested.

##### *Selected haematological parameters determination*

Full blood count was performed by Mindray BC-6800 auto-haematology analyzer. Blood in EDTA tubes were swirled gently and arranged on the analyzer racks. The analyzer was set to run after which the values of haemoglobin, red cell count and indices, platelet count and indices and white cell count and differentials were recorded.

### Data analysis

Data obtained from the study, were analyzed using Statistical Package for Social Sciences (SPSS) version 24.0.0.0 and a summary of results were presented using descriptive statistics of frequencies, means, medians, standard deviations and percentages. Variables were compared using independent sample t-test for normally distributed data and Mann-Whitney U-test for non-normally distributed data. Spearman’s rank correlation was used to determine correlation among the parameters. Probability value of P ≤ 0.05 was considered statistically significant.

## Results

### Characteristics of study participants

A total of 384 patients with type-2 diabetes who gave their informed consent were recruited for the study. This consisted of 258 (67.2%) females and 126 (32.8%) males. The ages of the participants ranged from 24 to 79 years with a mean age of 59.51 ± 12.831. The mean age of female participants was 58.34 ± 12.905 and that of male participants was 61.91 ± 12.616.

### Comparison of study parameters between male and female participants

The results obtained for the study participants is presented as Mean ± SD and Median (Q1-Q3) in the Table 1 below. There were no statistically significant differences (p < 0.05) in age and the measured parameters between male and female patients with diabetes. However, there were statistically significant differences in PLT, PCT and PDW-SD between male and female participants.

**Table 1:** A table showing the comparison of study parameters between male and female participants

Sex/Parameter	Female (N = 258)	Male (N = 126)	P-value
	Mean ± SD	Mean ± SD	
RBC (10^12/L)	4.29 ± 0.48	4.32 ± 0.55	0.813
HGB (g/dL)	11.54 ± 1.18	12.11 ± 1.23	0.065
HCT (%)	36.44 ± 3.64	38.02 ± 4.03	0.103
MPV (fL)	8.69 ± 0.94	9.21 ± 1.36	0.069
	**Median(Q1-Q3)**	**Median(Q1-Q3)**	
AGE	62(52–68)	63(49–71)	0.32
FBG (mmol/l)	8.6(6.8–11.1)	7.4(6.1–8.9)	0.1
HbA1c (%)	7.7(6.9–8.7)	7.4(6.6–9.1)	0.807
WBC (10^9/L)	5.08(4.02–6.28)	5.54(3.85–6.48)	0.68
NEU (10^9/L)	1.6(0.94–2.49)	1.02(0.36–2.32)	0.311
LYM (10^9/L)	2.58(2.14–3.04)	2.52(2.05–3.43)	0.945
MON (10^9/L)	0.5(0.37–0.74)	0.59(0.45–0.71)	0.274
EOS (10^9/L)	0.05(0.03–0.07)	0.07(0.04–0.09)	0.088
BAS (10^9/L)	0.05(0.03–0.07)	0.06(0.04–0.1)	0.085
MCV (fL)	86.2(80.3–91.9)	87.8(84.3–94.4)	0.179
MCH (pg)	27.6(24.8–29.2)	28.5(26.7–29.8)	0.218
MCHC (g/dL)	31.6(31.2–32.4)	32(31.3–32.7)	0.22
RDW-SD (fL)	39.6(37-41.8)	39.4(37.4–42.5)	0.653
PLT (10^9/L)	254(233–303)	232(204–254)	0.006 *S
PCT (%)	0.23(0.2–0.26)	0.2(0.18–0.21)	0.017 *S
PDW-SD (fL)	19.8(16.1–20.8)	21.4(19.3–23)	0.009 *S

S-SIGNIFICANT

### General correlation of Glycated haemoglobin and FBG with selected haematological parameters

When the Glycated haemoglobin and FBG were correlated with the selected haematological parameters, there were statistically significant correlations between HbA1c and NEU (p < 0.013), PCT (p < 0.036) and PDW-SD (p < 0.002) but not with the other measured parameters (p > 0.05). There was also no statistically significant correlation between FBG and any of the hematological parameters.

### Correlation of Glycated haemoglobin with selected haematological parameters among the participants according to gender

With regards to female participants, there was negative correlations between HbA1c and RDW-SD (p < 0.041), MPV (p < 0.005) and PDW-SD (p < 0.000) which were statistically significant, while there were both positive and negative correlations between HbA1c and the other parameters which were not statistically significant (p > 0.05). Male participants on the other hand had negative correlation between HbA1c and NEU (p < 0.006), and positive correlation with PLT (p < 0.009) and PCT (p < 0.007) all of which were statistically significant but no statistically significant correlations were seen with the rest of the parameters (p > 0.05) which showed both positive and negative correlations (Table 2).

**Table 2:** A table of the correlation of Glycated haemoglobin and selected haematological parameters among the participants

Sex	HbA1c/Parameter	Correlation Coefficient	P-value
Female (N = 258)	WBC (10^9/L)	0.022	0.885
	NEU (10^9/L)	-0.070	0.639
	LYM (10^9/L)	-0.080	0.595
	MON (10^9/L)	0.150	0.315
	EOS (10^9/L)	-0.206	0.165
	BAS (10^9/L)	0.261	0.077
	RBC (10^12/L)	0.149	0.316
	HGB (g/dL)	0.045	0.763
	HCT (%)	0.069	0.646
	MCV (fL)	-0.093	0.534
	MCH (pg)	-0.150	0.316
	MCHC (g/dL)	-0.222	0.134
	RDW-SD (fL)	-0.299	0.041 *S
	PLT (10^9/L)	-0.233	0.115
	PCT (%)	0.017	0.908
	MPV (fL)	0.400	0.005 *S
	PDW-SD (fL)	0.581	0.000 *S
Male (N = 126)	WBC (10^9/L)	-0.285	0.188
	NEU (10^9/L)	-0.556	0.006 *S
	LYM (10^9/L)	0.254	0.243
	MON (10^9/L)	-0.064	0.773
	EOS (10^9/L)	-0.129	0.557
	BAS (10^9/L)	0.011	0.961
	RBC (10^12/L)	0.115	0.602
	HGB (g/dL)	-0.062	0.779
	HCT (%)	0.029	0.895
	MCV (fL)	-0.173	0.431
	MCH (pg)	-0.173	0.429
	MCHC(g/dL)	0.005	0.981
	RDW-SD (fL)	-0.097	0.660
	PLT (10^9/L)	0.533	0.009 *S
	PCT (%)	0.546	0.007 *S
	MPV (fL)	0.178	0.418
	PDW-SD (fL)	0.110	0.618

** S-SIGNIFICANT**

### Correlation of FBG with selected haematological parameters among the participants

Statistical significance in correlation was absent between FBG and haematological parameters of female participants. Male participants on the other hand recorded statistically significant correlation between FBG and NEU (p < 0.012) and BAS (p < 0.035), but no statistically significant correlation of FBG with the other parameters (p > 0.05).

### Comparison of FBG and haematological parameters between Glycated haemoglobin groups

The results of Glycated haemoglobin were grouped into Group A (HBA1c < 7) and Group B (HBA1c ≥ 7). FBG and haematological parameters results between these groups were then compared. There were significant differences between the two HbA1c groups in FBG (increased in group B than A), NEU and RDW-SD (increased in group A than B), MPV and PDW (increased in group B than A). The other parameters most of which were increased in group A as compared to group B were however not statistically different between the two HbA1c groups (p > 0.05) (Table 3).

**Table 3:** A table showing the comparison of FBG and haematological parameters between the Glycated haemoglobin groups

**HbA1c Group/Parameter**	**Group A** **HbA1c < 7 (N = 121)**	**Group B** **HbA1c ≥ 7 (N = 263)**	**P-value**
	Mean ± SD	Mean ± SD	
Age	61.23 ± 7.55	58.73 ± 14.64	0.454
FBG (mmol/l)	7.52 ± 1.75	9.58 ± 3.62	0.014 *S
RBC (10^12/L)	4.30 ± 0.47	4.30 ± 0.52	0.985
HGB (g/dL)	11.92 ± 1.33	11.64 ± 1.17	0.371
HCT (%)	37.05 ± 3.96	36.92 ± 3.79	0.897
MCV (fL)	86.50 ± 7.65	86.51 ± 8.36	0.997
HbA1c Group/Parameter	Group A HbA1c < 7 (N = 121)	Group B HbA1c ≥ 7 (N = 263)	P-value
	**Median(Q1-Q3)**	**Median(Q1-Q3)**	
WBC (10^9/L)	5.78(4.08–6.72)	4.94(3.84–5.81)	0.217
NEU (10^9/L)	2.15(1.26–3.11)	1.36(0.51–2.35)	0.029 *S
LYM (10^9/L)	2.54(1.92–3.17)	2.59(2.22–3.17)	0.515
MON (10^9/L)	0.48(0.4–0.68)	0.53(0.42–0.74)	0.51
EOS (10^9/L)	0.07(0.05–0.09)	0.04(0.03–0.08)	0.075
BAS (10^9/L)	0.04(0.02–0.08)	0.06(0.03–0.08)	0.283
MCH (pg)	28.6(26.85–29.8)	27.45(25.03–29.18)	0.237
MCHC (g/dL)	32.05(31.28–33.33)	31.7(30.98–32.38)	0.057
RDW-SD (fL)	40.55(38.4-42.95)	38.9(36.25-41)	0.046 *S
PLT (10^9/L)	239.5(204-304.75)	246.5(225-286.25)	0.523
PCT (%)	0.2(0.18–0.23)	0.22(0.2–0.26)	0.056
MPV (fL)	8.4(7.78–8.98)	9(8.3–9.68)	0.032 *S
PDW-SD (fL)	19.2(15.68–20.83)	20.5(19.28–22.25)	0.013 *S

**S-SIGNIFICANT**

## Discussion

This study included patients with type 2 diabetes mellitus with mean age of 59.1 ± 12.831 which are in line with the global type-2 diabetes prevalence estimates of ages 20 to 79 years [1, 6]. The study also obtained a female proportion of 67.2% and male proportion of 32.8% with corresponding mean ages of 58. 34 ± 12.905 and 61.9 ± 12.616 respectively, similar to other studies [7, 10, 11]. When comparisons of FBG, HbA1c and selected hematological parameters by gender were carried out there were statistically significant differences in PLT, PCT and PDW-SD between male and female participants. This finding support the assertion that clinically, elevated platelet counts are frequently seen in diabetics with a long duration of disease and may be associated with the pathogenesis of vascular diseases in diabetes. However, no statistically significant differences were seen among the other parameters (Table 1). It was also seen that male participants had higher values of some parameters compared to female counterparts as seen in other studies [12, 13].

This study also obtained lower HbA1c levels in males [7.4 (6.6–9.1)] than females [7.7 (6.9–8.7] and with regards to FBG, this study recorded a mean value of 8.6mmol/l (6.8–11.1) among females and 7.4mmol/l (6.1–8.9) among males with no significant difference. When HBA1c were correlated with the selected haematological parameters, HbA1c significantly correlated with Neu, PCT, MPV and PDW-SD (levels of these parameters tend to increase in unregulated diabetic patients). Similar statistically significant correlations were observed by Demirtas et al., (2015) between HbA1c and PCT, MPV and PDW-SD [7].

With gender correlations, the female participants recorded significant statistical correlations between HbA1c and RDW-SD, MPV and PDW-SD (an indication of elevation of platelets and their indices in hyperglycaemia), while correlations with the other parameters were not statistically significant (p > 0.05). Male participants on the other hand had statistically significant correlation between HbA1c and Neu, PLT and PCT only (Table 2). It may be worthy to note that although not significant, the correlation between HbA1c and HGB was a negative one which is similar to the result of Panda & Ambade (2018) on the correlation between HbA1c and HGB [12].

When FBG was correlated with selected hematological parameters, no statistically significant correlation was recorded. But when HBA1c results were grouped into two (HbA1c < 7.0 and HbA1c ≥ 7.0), it was observed that 68.6% of the participants had their HbA1c levels at or above 7.0%, while a smaller proportion of 31.4% had their HbA1c levels below 7.0% as seen in similar studies [7]. When the two groups were compared with the other parameters, there were significant differences in FBG, Neu, RDW-SD, MPV and PDW between the two HBA1c groups (Table 3). The parameters that showed significant differences are known to give fluctuating levels in diabetes, particularly during unregulated diabetes. Furthermore, the finding of higher FBG in diabetes patients with higher HbA1c levels buttress the findings of Rohlfing et al., (2002) that there is a predictable relationship between plasma glucose concentration and the levels of HbA1c which is a probable indicator of poor glucose control among patients with diabetes [14].

## Conclusion

The findings of this current research showed significant correlation between Glycated haemoglobin and some selected haematological parameters (NEU, PCT and PDW-SD) an indication of the effect of poor glycaemic control on these haematological parameters. The findings obtained in this study should make measurement of routine haematological parameters a cost-effective means of predicting poor glucose regulation in patients with diabetes. The results also show that a large proportion of patients with diabetes are not properly managing their blood glucose concentration well a sign of poor glycaemic control among these patients.

### Limitations

There was limited time to carry out the study so some patient parameters such as blood pressure, lipid profile that could have helped with the study outcome could not be measured in this study.

### Electronic supplementary material

Below is the link to the electronic supplementary material.

Supplementary Material 1

Supplementary Material 2

## Data Availability

The datasets used and/or analyzed during the current study are available from the corresponding author on reasonable request.

## Abbreviations

EDTA: Ethylenediaminetetraacetic acid
EOS: Eosinophil
FBG: Fasting Blood Glucose
HbA1c: Glycated haemoglobin
HBG: Haemoglobin
HCT: Haematocrit
MPV: Mean platelet volume
NEU: Neutrophil
PCT: Plateletcrit
PDW: Platelet distribution width
PLT: Platelet
RBC: Red blood cell
RDW: Red cell distribution width
SD: Standard deviation
SPSS: Statistical package for Social Sciences
WBC: White blood cell
WHO: World Health Organization
