# Supplementary figures and images for: Comparative analysis of glycated haemoglobin, fasting blood glucose and haematological parameters in Type-2 diabetes patients
Source: BMC Res Notes. 2023 Oct 5;16:256. doi: 10.1186/s13104-023-06520-x (PMC10557280; doi:10.1186/s13104-023-06520-x)

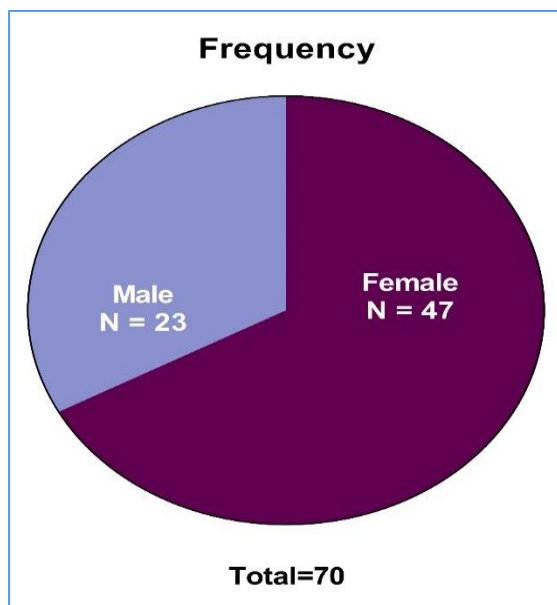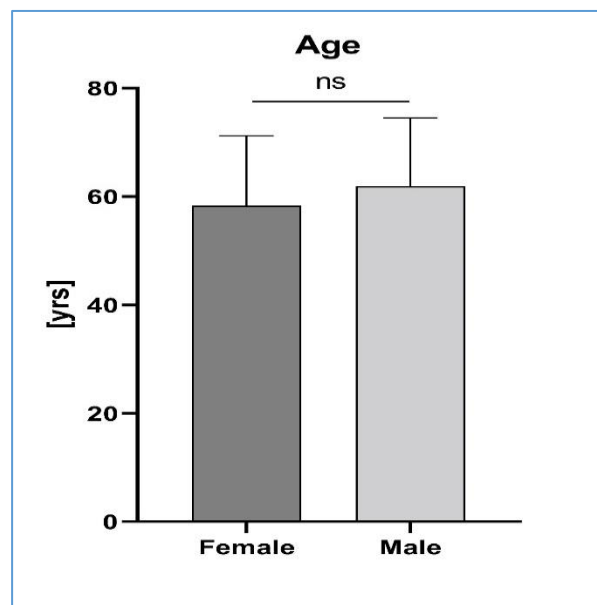

**Figure S1: A figure of the demographic characteristics of study participants**

Supplement: Supplementary file 1 — Supplementary Material 1 [file 13104_2023_6520_MOESM1_ESM.pdf]
