# Supplementary material for: Comparative analysis of glycated haemoglobin, fasting blood glucose and haematological parameters in Type-2 diabetes patients
Source: BMC Res Notes. 2023 Oct 5;16:256. doi: 10.1186/s13104-023-06520-x (PMC10557280; doi:10.1186/s13104-023-06520-x)
Supplement: Supplementary file 2 — Supplementary Material 2 [file 13104_2023_6520_MOESM2_ESM.docx]

**Table S1: A table of general correlation of Glycated haemoglobin with selected haematological parameters.**

| **HbA1c/Parameter** | **Correlation Coefficient** | **P-value** |
| --- | --- | --- |
| **WBC (10^9/L)** | -0.125 | 0.303 |
| **NEU (10^9/L)** | -0.295 | 0.013 *S |
| **LYM (10^9/L)** | 0.064 | 0.596 |
| **MON (10^9/L)** | 0.067 | 0.580 |
| **EOS (10^9/L)** | -0.194 | 0.108 |
| **BAS10^9/L)** | 0.173 | 0.152 |
| **RBC (10^12/L)** | 0.144 | 0.236 |
| **HGB (g/dL)** | -0.014 | 0.909 |
| **HCT (%)** | 0.068 | 0.574 |
| **MCV (fL)** | -0.147 | 0.226 |
| **MCH (pg)** | -0.193 | 0.109 |
| **MCHC (g/dL)** | -0.157 | 0.195 |
| **RDW-SD (fL)** | -0.231 | 0.054 |
| **PLT (10^9/L)** | 0.082 | 0.498 |
| **PCT (%)** | 0.251 | 0.036 *S |
| **MPV (fL)** | 0.279 | 0.019 *S |
| **PDW-SD (fL)** | 0.365 | 0.002 *S |

**S-SIGNIFICANT**

**Table S2: A table of general correlation of FBG with selected haematological parameters**

| **FBG/Parameter** | **Correlation Coefficient** | **P-value** |
| --- | --- | --- |
| **WBC (10^9/L)** | 0.077 | 0.529 |
| **NEU (10^9/L)** | -0.041 | 0.736 |
| **LYM (10^9/L)** | 0.181 | 0.134 |
| **MON (10^9/L)** | -0.028 | 0.819 |
| **EOS (10^9/L)** | -0.140 | 0.248 |
| **BAS (10^9/L)** | 0.195 | 0.106 |
| **RBC 10^12/L)** | -0.116 | 0.338 |
| **HGB (g/dL)** | -0.145 | 0.230 |
| **HCT (%)** | -0.154 | 0.205 |
| **MCV (fL)** | -0.100 | 0.408 |
| **MCH (pg)** | -0.070 | 0.562 |
| **MCHC (g/dL)** | -0.028 | 0.815 |
| **RDW-SD (fL)** | -0.080 | 0.511 |
| **PLT (10^9/L)** | 0.11 | 0.335 |
| **PCT (%)** | 0.137 | 0.258 |
| **MPV (fL)** | 0.020 | 0.867 |
| **PDW-SD (fL)** | 0.062 | 0.610 |

**Table S3: A table showing the correlation of FBG with selected haematological parameters among the participants according to gender.**

| **Sex** | **FBG/Parameter** | **Correlation Coefficient** | **P-value** |
| --- | --- | --- | --- |
| **Female (N=258)** | **WBC (10^9/L)** | 0.253 | 0.086 |
|  | **NEU (10^9/L)** | 0.195 | 0.188 |
|  | **LYM (10^9/L)** | 0.201 | 0.175 |
|  | **MON (10^9/L)** | 0.084 | 0.576 |
|  | **EOS (10^9/L)** | -0.080 | 0.593 |
|  | **BAS (10^9/L)** | 0.163 | 0.273 |
|  | **RBC (10^12/L)** | -0.142 | 0.342 |
|  | **HGB (g/dL)** | -0.188 | 0.206 |
|  | **HCT (%)** | -0.206 | 0.165 |
|  | **MCV (fL)** | -0.094 | 0.531 |
|  | **MCH (pg)** | -0.025 | 0.866 |
|  | **MCHC (g/dL)** | 0.090 | 0.548 |
|  | **RDW-SD (fL)** | -0.077 | 0.607 |
|  | **PLT (10^9/L)** | -0.051 | 0.732 |
|  | **PCT (%)** | 0.025 | 0.870 |
|  | **MPV (fL)** | 0.101 | 0.501 |
|  | **PDW-SD (fL)** | 0.123 | 0.411 |
| **Male (N=126)** | **WBC (10^9/L)** | -0.231 | 0.288 |
|  | **NEU (10^9/L)** | -0.515 | 0.012 *S |
|  | **LYM (10^9/L)** | 0.216 | 0.323 |
|  | **MON (10^9/L)** | -0.191 | 0.383 |
|  | **EOS (10^9/L)** | -0.081 | 0.713 |
|  | **BAS (10^9/L)** | 0.442 | 0.035 *S |
|  | **RBC (10^12/L)** | -0.030 | 0.891 |
|  | **HGB (g/dL)** | 0.059 | 0.789 |
|  | **HCT (%)** | 0.154 | 0.484 |
|  | **MCV (fL)** | 0.020 | 0.927 |
|  | **MCH (pg)** | -0.087 | 0.694 |
|  | **MCHC (g/dL)** | -0.258 | 0.234 |
|  | **RDW-SD (fL)** | -0.080 | 0.718 |
|  | **PLT (10^9/L)** | 0.250 | 0.250 |
|  | **PCT (%)** | 0.092 | 0.677 |
|  | **MPV (fL)** | 0.047 | 0.830 |
|  | **PDW-SD (fL)** | 0.225 | 0.302 |

**S-SIGNIFICANT**
